# St. Thomas's Hospital Armorial Bearings

**Published:** 1924-04

**Authors:** Charles A. H. Franklin

**Affiliations:** Hon. Sec. and Joint Hon. Treas. St. Thomas's Hospital Arms Fund


### St. Thomas's Hospital Armorial Bearings.

SIR,—I have read with considerable amazement your paragraph entitled "A Little Question of Heraldry," in your March issue. As this is far from being a correct exposition of the matter in question, I shall be very grateful if you will allow me fully to explain the position and put the real facts before your readers. Curiously enough, your issue appeared almost simultaneously with the December-January number of the *St. Thomas's Gazette*, in which the whole matter is dealt with in full. From it I reproduce the following :—

It is probably known to most St. Thomas's men past and present that for some years St. Thomas's Hospital has been using an heraldic device supposed to be the arms of the hospital. Eighteen months ago the writer being unable to find any authority for this device, he and the Secretary (G. Q. Roberts, C.B.E., M.A.) spent some time in examining the hospital charters and other documents to see whether the arms had ever been granted by Edward VI. or subsequently, but the search was negative. H.M. College of Arms was then approached upon the subject, and a report came (per Mr. G. Ambrose de Lisle Lee, C.B., Norroy King of Arms, now Clarenceux King of Arms) to the effect that the so-called "arms" were of no authority. The matter had also been gone into some 25 years ago by the late Dr. G. Marshall, LL.D., a then officer at the College, with the same result. It came as rather a shock to learn that St. Thomas's Hospital possessed no legal right to arms at all, but that the device used was entirely fictitious and a mere unauthorised assumption !

As the result of an interview between the Hon. Sir Arthur Stanley, G.B.E., Treasurer, and the writer, I was given authority by the Treasurer in writing to arrange for St. Thomas's Hospital to have a grant of arms by Letters Patent from the Crown to legalise the "arms" used, and to raise a fund sufficient to defray the fees and Stamp Duty payable upon the patent. So great and so ready was the response to this private appeal, that within a few days the entire sum was raised, and sufficient over to obtain in addition a fine painting of the arms upon vellum. . . . The total subscribed was £95 16s. Although all the fees were raised by generous private subscriptions and departmental contributions and the Petition presented for signature so long ago as April last, the matter is still held up, and some subscribers have been asking why this is so, and what has become of the money."

As Honorary Secretary and Treasurer of the fund, I felt that I owed the subscribers an explanation of the delay and an assurance that I had done and am doing all that I possibly could. Taking your paragraph point by point, there is no rashness in declaring that the "device" used by St. Thomas's is bogus: I have already referred to the report of H.M. Officers of Arms, the sole arbiters on all matters of Coat-Armour in England. The legality of arms depends now, as it has always depended, upon their recognition by the Crown or the duly appointed Officers of the Crown. Arms which they do not recognise are invalid and absolutely worthless. The "arms" used by the Hospital are also "spurious," as they have never had any legitimate existence whatever. You complained that the phrase used is highly derisory. I cannot alter or twist facts.

As regards the raising of the fund, I reproduce the letter from the Treasurer, the Hon. Sir Arthur Stanley, G.B.E., C.B., M.V.O., in which he authorised the matter to be proceeded with :—"Feb. 24, 1923. Dear Mr. Franklin,—If you could manage to arrange for us to have the grant of these Armorial Bearings without any cost to the Hospital itself, I should be very grateful.—Yours faithfully, Arthur Stanley."

There is no question of raising money "to pay the Heralds' College fee for a new grant of arms." The fees (£66 10s., Stamp Duty £10) are merely the reimbursement of the Crown of the cost of issuing a concession, and the defrayment of the Stamp Duty which the Exchequer has imposed. The Crown requires that the matter shall pass through the Royal College of the Incorporated Heralds.

I did not ask the permission of the Governors to apply for the grant; as I have already shown, I received due authority to arrange the matter, and having raised the necessary sum, I laid the Petition before the Treasurer for signature. The matter has never been before the Governors—of whom there are about 300—at all. Your statement that the Governors failed to see the necessity for the grant (a confirmatory grant) is already disposed of, as the matter has never been before the Governors. The few whom I approached gave most generous support. Investigation has found that down to 1752, at any rate, the Hospital was using the Arms of the City of London alone ("argent a cross gules, in the first quarter and sword erect of the same") two hundred years after Edward VI.'s Charter! There is no such thing as a prescriptive right to Arms in England, and there are no old families with unimpeachable Arms of which the College has no record. Arms that are not recorded in one of H.M. Offices of Arms are *ipso facto* non-existent.

As regards the ultimate destination of the money raised, it can only be used for the specific purpose for which it was raised, or be returned in full to subscribers. There is no possibility of the money being used for a hospital appliance, however useful. In any case the full and written consent to such a course from every subscriber would be necessary first, which is unobtainable.

Charles A. H. Franklin,
Hon. Sec. and Joint Hon. Treas. St. Thomas's Hospital Arms Fund.

[We were of course aware that in England a right to arms cannot be acquired by user or prescription. We employed the word "prescription" in its colloquial sense.—Ed., H. & H. R.]

